# Our New York Letter

**Published:** 1887-05

**Authors:** 


### Correspondence.

### OUR NEW YORK LETTER.

EXPERT TESTIMONY—HOW IT SHOULD BE OBTAINED—TREATMENT OF OLD DISLOCATIONS OF THE ELBOW—A LARGE BRAIN TUMOR—BACTERIA IN NEW YORK WATER AND ICE, ETC.

*To the Editors of the Atlanta Medical and Surgical Journal:*

An address, entitled "Expert Testimony," was lately delivered by Mr. St.Clair McKelway before the Society of Medical Jurisprudence. His remarks referred principally to the scandals and abuses arising from the present system of employing partisan experts who habitually contradict each other. After speaking of numerous remedies against this evil, he proposed the following plan: Mr. McKelway believed that the great power which our equity system gives to courts of original jurisdiction should be sufficient to authorize judges to appoint a single expert, and obtain his ideas about any subject under consideration. It is for any judge to say, if he wishes to exercise his power, whether he has heard sufficient testimony on any subject. He can say, "I have heard sufficient on that subject—call the witness on the other side," and both parties in the trial would have to submit to this ruling. But this, however, is seldom the case. He proposed that the courts summon a single expert who could be cross-examined by the lawyers on either side. The expert would, he believed, be induced to give faithful testimony, knowing that his pay was to come from the court and not from either party in the trial. His opinion was that a man will generally educate himself to believe that theory to be correct which will most favor the interests of his retainer.

A very excellent steel engraving of the late Dr. Gunning S. Bedford has been presented to the Academy of Medicine by its President, Dr. A. Jacoby. In making this presentation, Dr. Jacoby spoke of the important services rendered by the deceased and the enviable character he bore. He deserved the credit of founding the first obstetrical clinic in this country, and was especially noted for his kindly attentions to the poor. Dr. Bedford's granduncle was a Revolutionary patriot and the constitution bears his signature. After being educated in the law, young Bedford had his attention turned to medicine by listening to a lecture on the circulation of the blood delivered by Dr. John D. Goodman. He studied both here and abroad and held professorships in several medical schools. His name also is connected with the founding of the New York University Medical College in 1841. Subsequently, he presented this college with a very valuable anatomical museum of his own collecting. At the time of his death, he was spoken of by the Academy as "a most illustrious and devoted practitioner."

At a recent meeting of the Surgical Society, Dr. L. A. Stinson read a paper "On the Treatment of Old Dislocations of the Elbow." During his service at Bellevue Hospital, a case came under his care presenting a backward dislocation of both bones of the forearm, with a fracture of the external epicondyle of five months' standing. The great degree of disability existing was relieved by an arthrotomy, the bony obstructions being removed. A recurrence of the dislocation occurred, however, and in two months a second operation was done. This time he obtained flexion to a right angle and almost complete extension, but seven months later, during the doctor's absence from New York, the joint became completely ankylosed at a right angle. He had noticed in these dislocations the following peculiar changes: A new growth of bone at the back of the external condyle, probably due to a stripping up of the periosteum; Filling of the greater sigmoid cavity by a mass of fibrous tissue; an exaggerated growth of the epipysis of the humerous due to removal of pressure normally exerted upon it by the opposing articular surfaces. The neck of the radius might elongate from the same cause. Partial fracture of the radial head and coronoid process might interfere with reduction. In cases of over one month duration, strong adhesions will have formed which must be divided before reduction could be accomplished, and even then the bony deformity might seriously interfere with the functions of the joint. The treatment, he remarked, must necessarily differ from that employed in recent cases, even if we could employ sufficient force to rupture the adhesions and reduce the displacement. In elderly patients reductions should be attempted after rupturing or dividing subcutaneously the adhesions, and failing in this the elbow should be flexed to a right angle and allowed to ankylose. In young patients, when reduction fails, he would recommend arthrotomy, and should this be unsuccessful a resection could be made. Arthrotomy should be postponed until all inflammatory reaction from the recent injury or attempts at reduction has subsided. The younger the patient (under 15 years) the greater was the probability that excision would be required. In old, incomplete, outward dislocations, arthrotomy, he said, offered the only hope.

During the discussion which followed, Dr. R. F. Weir said that as some of the cases did well with time, surgeons had often been led to leave them to nature. He had lately concluded that we could properly interfere at an earlier period than was generally thought safe, and that after failing in reduction we should operate, sewing up the rent in the capsule produced by the original injury. The early operation, he believed, gave a better prognosis.

Dr. A. G. Gerster remarked that the periosteum might be stripped off the external condyle and the interval between it and the bone would fill with blood-clot, which then became organized into connective tissue and bone. All his cases had been complicated with fracture and the so-called "gun-stock" deformity was well marked. He believed arthrotomy to be perfectly proper at the present day under the antiseptic system, and it would be wrong not to interfere when attempted reduction failed, early or late. The operator, he said, should, however, be thoroughly versed in antiseptics, or the patient's life would be risked. The

President, Dr. C. McBurney, favored early operation in these cases, not later than two or three months after the accident.

Dr. Weir exhibited a tumor which he had removed from the posterior occipital region of the brain. It was the largest brain tumor that had been removed by a surgical operation. A complete history of the case was promised for a future meeting of the society.

Dr. T. Mitchell Prudden, Director of the Pathological Laboratory of the College of Physicians and Surgeons, has been making an elaborate series of experiments on the bacteria in water and in New York's ice supply. This city is supplied in small part with ice from several small ponds and lakes which are almost free from animal contamination. But the great bulk of our ice comes from the Hudson river, which is known to be sewage-polluted. Dr. Prudden found that the freezing process, even when prolonged, did not kill the typhoid or the suppuration bacillus. The idea that water purified itself in freezing was only true to a limited extent. The greatest number of bacteria were found in bubbly or snow-ice, and the transparent contained less. Alternate freezing and thawing caused the greatest destruction of bacteria. Neither the typhoid nor the suppuration bacillus had been seen in Hudson river ice, yet it was known that the former are discharged in enormous numbers with all typhoid dejections, and as these flow into the sewers which empty into the river their presence in the ice is highly probable. Dr. Prudden recommended that the ice-fields be placed under the supervision of the State Health Board, and also that the bubbly and snow-ice be reserved for cooling purposes only and not used for drinking.

A. F.

*New York,, April 1887.*

